# Flamingos and drought as drivers of nutrients and microbial dynamics in a saline lake

**DOI:** 10.1038/s41598-017-12462-9

**Published:** 2017-09-22

**Authors:** Gema L. Batanero, Elizabeth León-Palmero, Linlin Li, Andy J. Green, Manuel Rendón-Martos, Curtis A. Suttle, Isabel Reche

**Affiliations:** 1 Departamento de Ecología e Instituto del Agua, Universidad de Granada, 18071 Granada, Spain; 2 Department of Natural Resources, Faculty of Geo-information Science and Earth Observation, University of Twente, 7500 AE Enschede, The Netherlands; 3 Departamento de Ecología de Humedales, Estación Biológica de Doñana, EBD-CSIC, 41092 Sevilla, Spain; 4 Reserva Natural Laguna de Fuente de Piedra, Consejería de Medio Ambiente y Ordenación del Territorio, Junta de Andalucía, Apartado 1, 29520 Fuente de Piedra, Málaga, Spain; 5 Departments of Earth, Ocean & Atmospheric Sciences, Microbiology & Immunology, and Botany, and the Institute for the Oceans and Fisheries, University of British Columbia, Vancouver, BC V6T 1Z4 Canada

## Abstract

Waterbird aggregations and droughts affect nutrient and microbial dynamics in wetlands. We analysed the effects of high densities of flamingos on nutrients and microbial dynamics in a saline lake during a wet and a dry hydrological year, and explored the effects of guano on prokaryotic growth. Concentrations of dissolved organic carbon, total phosphorus and total nitrogen in the surface waters were 2–3 fold higher during the drought and were correlated with salinity. Flamingos stimulated prokaryotic heterotrophic production and triggered cascading effects on prokaryotic abundance, viruses and dissolved nitrogen. This stimulus of heterotrophic prokaryotes was associated with soluble phosphorus inputs from guano, and also from sediments. In the experiments, the specific growth rate and the carrying capacity were almost twice as high after guano addition than in the control treatments, and were coupled with soluble phosphorus assimilation. Flamingo guano was also rich in nitrogen. Dissolved N in lake water lagged behind the abundance of flamingos, but the causes of this lag are unclear. This study demonstrates that intense droughts could lead to increases in total nutrients in wetlands; however, microbial activity is likely constrained by the availability of soluble phosphorus, which appears to be more dependent on the abundance of waterbirds.

## Introduction

Since Hutchinson’s seminal work^1^ on the importance of guano on marine productivity, many studies have analyzed the inputs of nutrients associated with waterbird feces in inland waters^2–6^. This process is termed guanotrophication^7^ and appears to be particularly important in arid regions^8^, systems with long water residence times^9^ and inland waters used as roosts by non-breeding birds or as breeding sites by colonial waterbirds, where birds import nutrients from foraging areas^4,5,10–12^. Guanotrophication affects wetland quality and primary productivity, as shown in studies by Kitchell *et al*.^3^ in which chlorophyll increased with the density of geese, and in experiments with phytoplankton^13^. On the other hand, large waterbirds cause sediment bioturbation with consequences for nutrient release and methane fluxes^14,15^. Although bacterial production and diversity are known to change under pulses of nutrients^16–18^, no studies have directly addressed changes in aquatic microbial communities associated with guanotrophication and sediment bioturbation by waterbirds, despite the key role of microbial processes in biogeochemical cycles and greenhouse gas fluxes in wetlands and lakes.

Insights into the effects of waterbirds on microbial communities are crucial to enable integrative wetland management and the measures required to support waterbird diversity and their effects on guanotrophication, nutrient recycling, microbial-derived processes and water quality in general^9,19^. This is even more important given the consequences of climate change that is affecting hydrological regimes in contrasting ways in different biomes^20^. Projected changes suggest an increase in wetland areas in tropical or polar latitudes; whereas temperate and Mediterranean wetlands and lakes may reduce their hydroperiod or dry out completely^21,22^, with major implications for species diversity^23^. In the Mediterranean biome, extended droughts could reduce runoff and increase evaporation, causing salinization of many wetlands and lakes^22,24,25^. This reduction in wetland surface or in hydroperiod length may promote the overcrowding of waterbirds in arid or semiarid regions during breeding or wintering periods^8,9^, which may reduce water quality through guano inputs and sediment bioturbation^2^. Similar effects may be caused by other kinds of global change such as increasing water extraction or the increase in bird populations resulting from exploitation of anthropogenic habitats (e.g. agricultural fields or fish farms) or protection from disturbance and hunting. For example, numbers of flamingos and other wading birds have increased markedly in southern Spain in recent decades due to increased protection and exploitation of artificial habitats, such as ricefields and fish ponds^26,27^.

Flamingos represent a major fraction of the waterbird biomass in saline lakes in Africa^28,29^, and have profound effects on the limnology of Andean salt lakes^30^. In the Western Mediterranean region, their abundance and movements are particularly well monitored^31–35^. Here, we studied the influence of flamingos on nitrogen, phosphorus, and microbial dynamics in a saline lake that holds the largest flamingo colony in the Western Mediterranean. Flamingos can increase N and P concentrations by guano inputs from mass aggregations during breeding periods, and by sediment bioturbation during feeding and trampling in the lake. We hypothesized that in this lake (i) dissolved nitrogen and phosphorus concentrations would be related with flamingo abundance due to guanotrophication and sediment bioturbation and used correlation and regression analysis to test it; (ii) dissolved nutrients from guano would boost heterotrophic prokaryotic production affecting microbial dynamics and performed two experiments and cross-correlations to test it; (iii) drought would concentrate nutrients and microbial cells by evaporation, and therefore intensify the effects of flamingos and we compared the nutrient and microbial dynamics in two well-contrasted (wet and dry) hydrological years to test it. Finally, we discuss the implications of our findings given ongoing climate change and the effects of conservation policies on waterbird densities.

## Material and Methods

### Study site, water level and flamingo abundance

**Figure 1:**
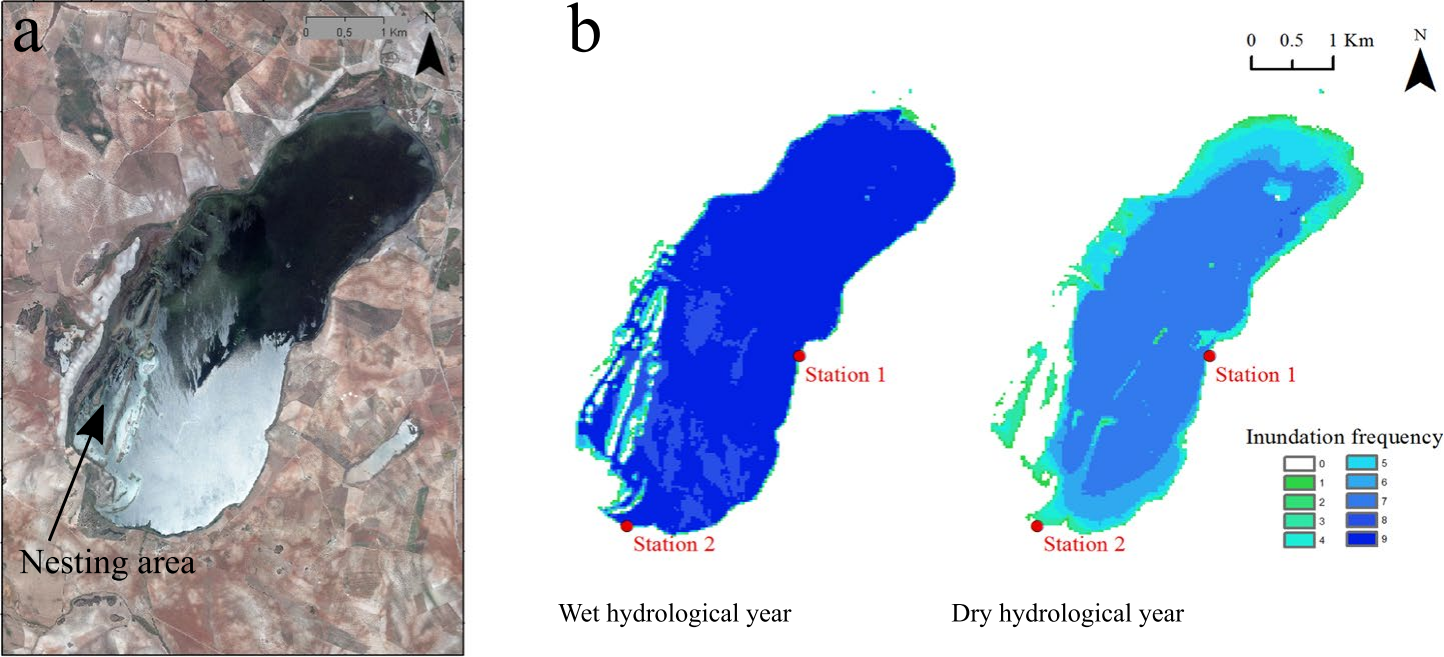
Images of the saline lake studied (Fuente de Piedra, Málaga, Spain). (**a**) Orthophoto (http://ws041.juntadeandalucia.es/medioambiente/dlidar/index.action) showing the flamingo nesting area, and (**b**) frequency of inundation area of the lake obtained from Landsat imagery (https://earthexplorer.usgs.gov/) for the wet and the dry hydrological years. Images of similar dates were used to calculate the inundation frequency for each pixel, which ranged from 0 (i.e. lake surface dry in all 9 images) to 9 (i.e. inundated in all 9 images). The location of the two sampling stations is shown in red dots.

This study was performed in Fuente de Piedra, an athalassohaline lake located in an endorheic basin of karstic origin in the south of Spain (37° 6′ N, 4° 44′ W) (Fig. 1a). Its hydrology is mainly linked to inputs from rainfall, two intermittent streams (Santillán and Humilladero) and ground water, and outputs mainly by evaporation^36,37^. This large saline lake covers a maximum area of 1350 ha and supports one of the most important breeding colonies of the greater flamingo (*Phoenicopterus roseus*) in the Western Mediterranean^34,38^. Breeding adults fly within a radius of 350 km to feed in other wetlands, returning to feed their chicks at Fuente de Piedra^31,32,39^.

We sampled this lake during more than two hydrological years. The first hydrological year between September 2010 and August 2011 was considered to be wet as the lake was inundated for most time of the year (Fig. 1b); it was similar to the previous hydrological year that was only partially sampled. In contrast, the second hydrological year (i.e. from September 2011 to August 2012) was relatively dry because the lake dried out during the summer (Fig. 1b). Each hydrological year had two phases. The first phase comprises water filling during fall and winter (September-March); whereas, the second phase consists of water evaporation during spring and summer (April-August).

The different hydrological conditions cause intense changes in the water level of the lake^40,41^. The water level in Fuente de Piedra was measured daily using a limnigraph that registers water level variations on a paper graph through movements of a floating sensor that is located in an open shallow well in the lagoon. Changes in the inundation area were obtained with multi-temporal Landsat imagery. We collected for each hydrological year nine images with similar dates from the USGS EarthExplorer (https://earthexplorer.usgs.gov/). The nine images (for each hydrological year) were classified as water and non-water^42^ and then added up to calculate the inundation frequency for each pixel using ArcGIS 10.4 software. The resulting inundation frequency was ranged from 0 (i.e. the lake was dry in all 9 images) to a maximum of 9 (i.e. the lake was inundated in all 9 images).

Samples were taken from the water column (ca. 10 cm below the surface) at two stations (red dots in Fig. 1b). Station 1 is less affected by wind and turbidity and is a flamingo foraging area, whereas Station 2 is located nearer the nesting area and is more exposed to wind, evaporation and turbidity. Samples were collected biweekly from July 2010 to November 2012, except when the lake was dry from June to October of 2012.

Flamingos on the lake were counted one to seven times per week during the breeding period and monthly the rest of the year, using binoculars (8 × 30) and a spotting scope (20–60 × 60). Birds were counted one by one for small groups, or in groups of five individuals for big aggregations. The abundance of chicks during breeding was obtained from aerial pictures. Rainfall, water level and flamingo data were provided by the “Consejería de Medio Ambiente y Ordenación del Territorio” of Junta de Andalucía, Spain.

### Physico-Chemical analyses

Salinity, pH and temperature were measured using a multi-parameter probe (HANNA HI 9828). Total nutrient concentrations were measured in unfiltered water, while samples for dissolved nutrient analysis were filtered through 0.7 μm pore-size Whatman GF/F glass-fiber filters. Soluble reactive phosphorus (SRP), total phosphorus (TP) and total dissolved phosphorus (TDP) concentrations were measured using the molybdenum blue method^43^, the latter as SRP, after digestion with a mixture of potassium persulphate and boric acid at 120 °C for 30 min^44^. Total nitrogen (TN) and total dissolved nitrogen (TDN) were analysed by high–temperature catalytic oxidation^45^ using a total nitrogen analyzer (TNM-1, Shimazdu TOC-V CSH). Samples for dissolved organic carbon (DOC) were collected from surface waters in a combusted (>2 h at 500 °C) flask, filtered through pre combusted GF/F filters, acidified with phosphoric acid (final pH < 2) and stored at 4 °C in the dark until analysis. DOC concentration was measured by high-temperature catalytic oxidation in a Shimadzu total organic carbon (TOC) analyzer (Model TOC-V CSH). The instrument was calibrated using a four-point standard curve of potassium hydrogen phthalate. Samples were purged with phosphoric acid for 20 min to eliminate any dissolved inorganic carbon. Three to five injections were analysed for each sample.

### C, N and P content in chick flamingo guano

Fresh flamingo chick feces were collected during banding operations in the Fuente de Piedra colony, immediately frozen in liquid nitrogen and stored at −80 °C until analysis. Feces were thawed and classified as either pink or brown, reflecting differences in diet. Prior to analysis, feces were dried at 60 °C for at least 24 h to obtain dry weight. They were then diluted in a known volume of Milli-Q water, and TN, TDN, TP, TDP, TOC and DOC concentrations were determined as explained above.

Per capita N and P loads associated with flamingo guano were estimated as functions of body mass and the N and P contents per g of dry feces (Eq. 4 in Hahn *et al*.^4^). The average body mass of an adult flamingo male is 3579 g and of an adult female is 2525 g^46^, so we took 3052 g as average body mass for adults and assumed all feces produced were deposited in the lake. Daily N and P inputs were obtained by multiplying per capita excretion by flamingo abundance, and annual loadings by summing all daily values over the year. Lake volume was calculated by integrating the area beneath the hypsographic curve (i.e. surface area vs. water level)^47^.

### Biological analyses

Chlorophyll *a* concentrations were determined by collecting the particulate material from 100 to 700 ml of water by filtering through 0.7 μm pore-size Whatman GF/F glass-fiber filters, then extracting the filters with 95% methanol in the dark at 4 °C for 24 h^44^. Pigment absorption was measured using a Perkin Elmer UV-Lambda 40 spectrophotometer at wavelengths of 665 nm and 750 nm.

The abundances of prokaryotes (PA) and viruses (VA) were determined in triplicate samples using flow cytometry in a FACScalibur flow cytometer (excitation 448 nm), and analysed in bivariate plots of Side scatter (SSC) *vs*. FL1 (Green fluorescence)^48,49^. For PA, samples were collected and fixed with a mixture of 1% paraformaldehyde and 0.05% glutaraldehyde for 30 min in the dark at 4 °C, frozen in liquid nitrogen, and stored at −80 °C until analysis. In the laboratory, the samples were thawed and diluted ≥10-fold with Milli-Q water to avoid coincidence of cell counts. Samples were stained with a 10 μM DMSO solution of SYBR Green I (Molecular Probes) for 10 min in the dark. Yellow-green 0.92 μm latex beads (Poysciences) were used as an internal standard. For VA, samples were fixed with glutaraldehyde 0.5% for 15–30 min at 4 °C in the dark, flash frozen in liquid nitrogen and stored at −80 °C until analysis (Brussaard *et al*., 2010). Prior to analysis, samples were diluted ≥100-fold with TE-buffer pH 8.0 (10 mM Trishydroxymethyl-aminomethane; 1 mM ethylenediaminetetraacetic acid) to avoid the coincidence in virus particle counts. VA samples were stained with a working solution (1:200) of SYBR Green I (10,000X concentrate in DMSO, Molecular Probes) for 10 min in the dark and then kept at −80° until counting. Fluorescent microspheres (FluoSpheres carboxylate modified yellow-green fluorescent microspheres; 1.0 μm diameter) were added as an internal standard. Data were analyzed using BD CellQuest Pro software.

Prokaryotic heterotrophic production (PHP) was estimated from ^3^H-Leucine- incorporation following the microcentrifugation technique^50^. Three 1.5 ml replicates and two trichloroacetic acid (TCA)-killed blanks were prepared for each sampling station and day. In each sample, 5 μl of (4,5–^3^H)-L-Leucine was added to a final concentration of 54.6 nM and then incubated for 2–5 h at *in situ* temperature. Adding TCA at a final concentration of 10% terminated the incubations. In the laboratory, the samples were centrifuged (10 min at 14000 rpm), rinsed with 5% TCA, vortexed, and centrifuged again. For each sample, 1.5 ml of liquid scintillation cocktail (Ecoscint A) was added, and the radioactivity determined using an autocalibrated scintillation counter (Beckman LS 6000 TA).

### Experiments with guano

**Figure 2:**
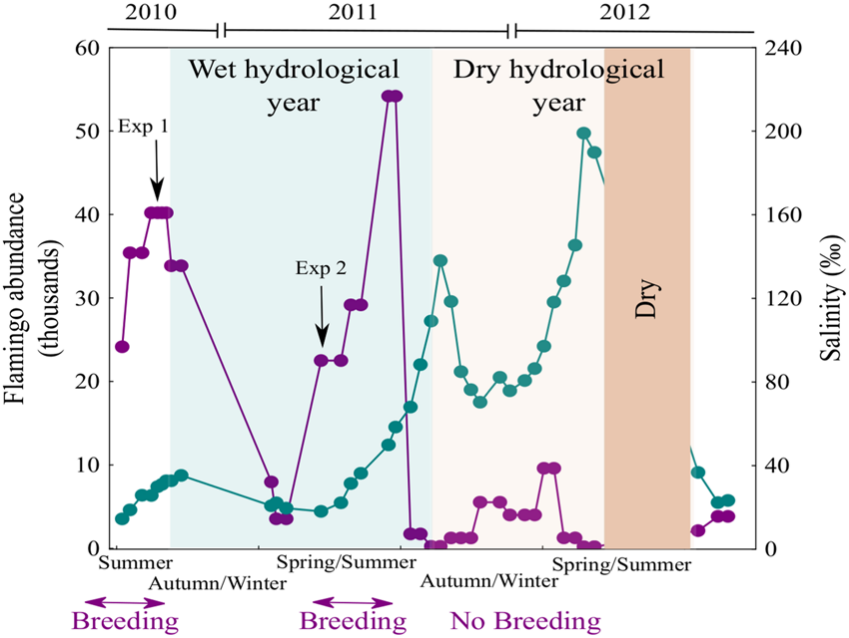
Changes in salinity and abundance of flamingos. Flamingo abundance is shown by the purple line and salinity by the blue-green line. The wet hydrological year is shaded in blue and the dry hydrological year in cream. The lake dried out completely during the period shaded in brown. The periods of flamingo breeding are marked on the x-axis.

To determine the effects of guano on prokaryote growth, we carried out two experiments in the laboratory using previously frozen feces from flamingo chicks collected during the ringing operations in August 2010. Chicks are fed with a secretion produced by their parents that fly to forage in other wetlands^39,51^ and represent most of the individuals present constantly in the lake during breeding periods. The first experiment (Exp.1) was carried out using lake water on October 10^th^ 2010 when there were approximately 34000 flamingos in the lake and a water level of 79 cm, and the second experiment (Exp. 2) was performed using lake water on May 5^th^ (2011 year) when there were approximately 23000 flamingos in the lake and a water level of 130 cm (Fig. 2). Each experiment consisted of two treatments and each treatment had three replicates. Controls consisted of 800 ml of 0.2-μm-filtered lake water, plus 200 ml of water filtered through a 0.8 μm pore-size Whatman GF/F filter that allowed most bacteria to pass through, but removed bactivorous flagellates. The + Guano treatment was the same as the control, with the addition of sterilized chick feces. All the experimental (1 litre Pyrex) bottles were incubated in the dark at *in situ* temperature using a culture chamber. From each treatment, triplicate subsamples were collected and prokaryotic abundance and production were quantified every 12 h to during the 84 h incubation period, and samples for dissolved nutrients were taken at the initial (t_0_) and final (t_f_) sampling time. We determined prokaryotic abundance and production, and the concentration of dissolved nutrients, as described above for lake samples. Changes in prokaryotic abundance and production throughout the incubations were fitted to exponential or logistic functions. The fits and growth parameters (specific growth rates and carrying capacities) were obtained using nonlinear estimations. More details on the estimation procedure can be found in Pulido-Villena & Reche^52^.

### Statistical Analysis

We performed multiple regression analysis to assess the main drivers of SRP and TDN including flamingo abundance (proxy for guano inputs) and water level (proxy for sediment bioturbation and evaporation) and correlation analyses were performed to assess the relationships between major nutrients and microbial variables with flamingos and salinity. Finally, we used a two-way cross-correlation to analyse the sequential effects of flamingos on microbial and nutrient components in more detail. The cross-correlation coefficient (k) represents the correlation between two time series (i.e., microbial and nutrient parameters [X] *vs*. flamingo abundance [Y]), where X is lagged forward or backward by (k, 10) observations. For this analysis we sampled every two weeks between August 2011 and May 2012 at station 1. All statistical analyses were performed using Statistica (v.7.0).

## Results

### Nutrient dynamics in the lake

The first hydrological year was wet with an annual rainfall of 563.3 mm, a salinity of ~30 ppt, water levels ranging from 44 cm to 130 cm, and an average lake surface area of 992 ha with more than 50000 flamingos during the breeding period (blue period in Fig. 2); it was similar to the previous hydrological year that was only partially sampled. In contrast, the second hydrological year was dry with an annual rainfall of 345.1 mm, a salinity that was always above 70 ppt, a water level that ranged from 52 cm to 0 cm when the lake dried up, an average surface area of 522 ha, and a maximum of only 12000 flamingos with no breeding (brown period in Fig. 2) (Table 1).

**Table 1:** Mean values and ranges (in parentheses) of basic physicochemical and biological variables in the two sampling stations during the wet and dry hydrological years.

Station Localization	Hydrological year	Rain (mm)	Temperature (°C)	Salinity (ppt)	pH	DOC (mmol-Cl^−1^)	TN (mmol-Nl^−1^)	TP (μmol-Pl^−1^)	TN:TP (Molar)	TDN (mmol-Nl^−1^)	SRP (μmol-Pl^−1^)	Chl a (μg/L)
Station 1 37°06′19.8″N 4°45′44.5″W	2010–2011 Wet	563.3	19.1 (8.3–29.4)	38.3 (18.1–88.3)	9.03 (8.04–9.94)	1.99 (1.07–4.72)	0.22 (0.08–0.48)	5.12 (1.38–11.52)	52 (30–93)	0.18 (0.06–0.48)	0.41 (0.18–1.61)	44.2 (2.4–111.9)
	2011–2012 Dry	345.1	17.6 (9.3–25.6)	112.5 (70.3–199.0)	8.96 (7.46–9.06)	6.19 (3.85–13.59)	0.60 (0.32–1.17)	13.82 (5.78–38.60)	49 (24–85)	0.28 (0.07–0.80)	0.57 (0.18–1.63)	44.9 (14.5–73.3)
Station 2 37°05′07.4″N 4°47′08.3″W	2010–2011 Wet	563.3	19.7 (8.2–33.5)	41.6 (21.4–91.8)	9.03 (8.04–9.94)	1.98 (1.00–4.73)	0.22 (0.06–0.49)	4.57 (1.59–10.20)	50 (32–79)	0.18 (0.06–0.46)	0.47 (0.18–1.54)	20.7 (3.9–50.0)
	2011–2012 Dry	345.1	19.7 (8.3–33.5)	109.9 (67.7–188.0)	8.26 (7.61–8.99)	6.03 (3.79–10.02)	0.57 (0.35–0.83)	11.40 (5.60–23.93)	55 (30–98)	0.28 (0.07–0.74)	0.90 (0.22–0.91)	58.4 (23.1–116.1)

**Figure 3:**
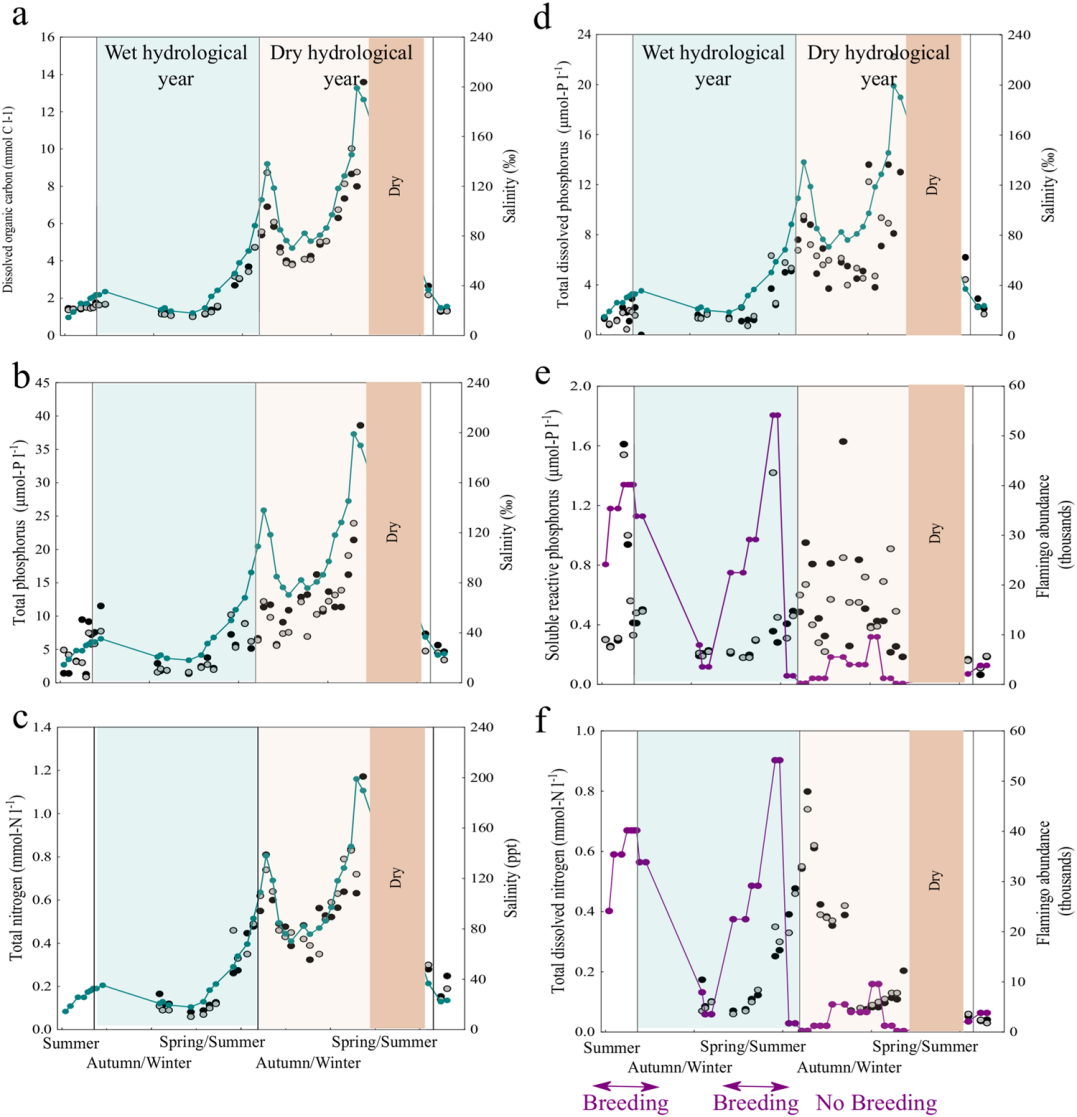
Nutrient dynamics during two hydrological years. Changes in the concentration of (**a**) dissolved organic carbon, (**b**) total phosphorus, (**c**) total nitrogen, (**d**) total dissolved phosphorus, (**e**) soluble reactive phosphorus and (**f**) total dissolved nitrogen at station 1 (black dots) and station 2 (grey dots). Salinity (blue-green line) or flamingo abundance (purple line) are shown for reference.

Nutrient concentrations varied dramatically over the course of the study. DOC concentration ranged more than an order of magnitude from 1.00 mmol-C l^−1^ to 13.59 mmol-C l^−1^, with the highest values during the dry year (Table 1). Changes in DOC concentration and salinity were tightly correlated (n = 73, r = 0.97, p < 0.0001) and coupled with the filling (autumn/winter) and evaporation (spring/summer) phases (Fig. 3a). The TP concentration varied almost 30-fold from 1.38 μmol-P l^−1^ to 38.60 μmol-P l^−1^ (Table 1), reached the maximum during the evaporation phase of the dry year, and consequently, was strongly correlated with salinity (n = 75, r = 0.79, p < 0.0001) (Fig. 3b). TN concentration varied 20-fold from 0.06 to 1.17 mmol-N l^−1^ (Table 1), reaching maximum values during the dry year. TN also showed a tight correlation with salinity (n = 65, r = 0.90, p < 0.0001) (Fig. 3c). TDP concentration varied from 0.73 to 22.23 μmol-P l^−1^, and reached its maximum during the dry year, coupled with salinity (Fig. 3d). As well as evapoconcentration, there was evidence that sediment interactions influenced TDP concentration, as TDP increased linearly as the water level (WL) decreased below 80 cm (TDP = 15.7–0.18 WL, r^2^ = 0.38, p < 0.001, Fig. S1 in supplementary material). In contrast, SRP varied from 0.18 to 1.63 μmol-P l^−1^ (Table 1), was not significantly correlated with salinity, and showed a first peak during the wet year, concomitant with a major increase in the abundance of flamingos (Fig. 3e). TDN concentrations also ranged more than ten-fold from 0.06 mmol-N l^−1^ to 0.80 mmol-N l^−1^ (Table 1), showed dynamics time-lagged with the abundance of flamingos, and was not influenced by salinity during the dry year (n = 30, r = 0.013, p = 0.944) (Fig. 3f). To explore in more detail the relevance of water level (indicative of sediment influence and evaporation) and guanotrophication by flamingos on SRP and TDN concentrations in the water, we performed multiple regression analysis (Table 2). Both variables significantly affected SRP concentrations, although the contribution of water level was stronger (higher partial correlation coefficient) than that of flamingo abundance. TDN concentration was also significantly affected by the water level, and not directly by the flamingo abundance.

**Table 2:** Results of multiple regression analyses to assess the influence of flamingo abundance and water level on the concentrations of soluble reactive phosphorus and total dissolved nitrogen.

Independent Variables	Soluble Reactive Phosphorus (μmol-P l^−1^)			Total Dissolved Nitrogen (mmol-N l^−1^)		
	*r*	*b*	p-*level*	*r*	*b*	p-*level*
Flamingos	0.368787	0.000007	0.002964	—	—	—
Water level (cm)	−0.422263	−0.004579	0.000746	−0.422129	−0.002525	0.000868
Intercept		0.681651	0.000000		0.390625	0.000000

The *r* values are partial correlation coefficients, *b* is the non-standardized regression coefficients.

### Microbial dynamics

**Figure 4:**
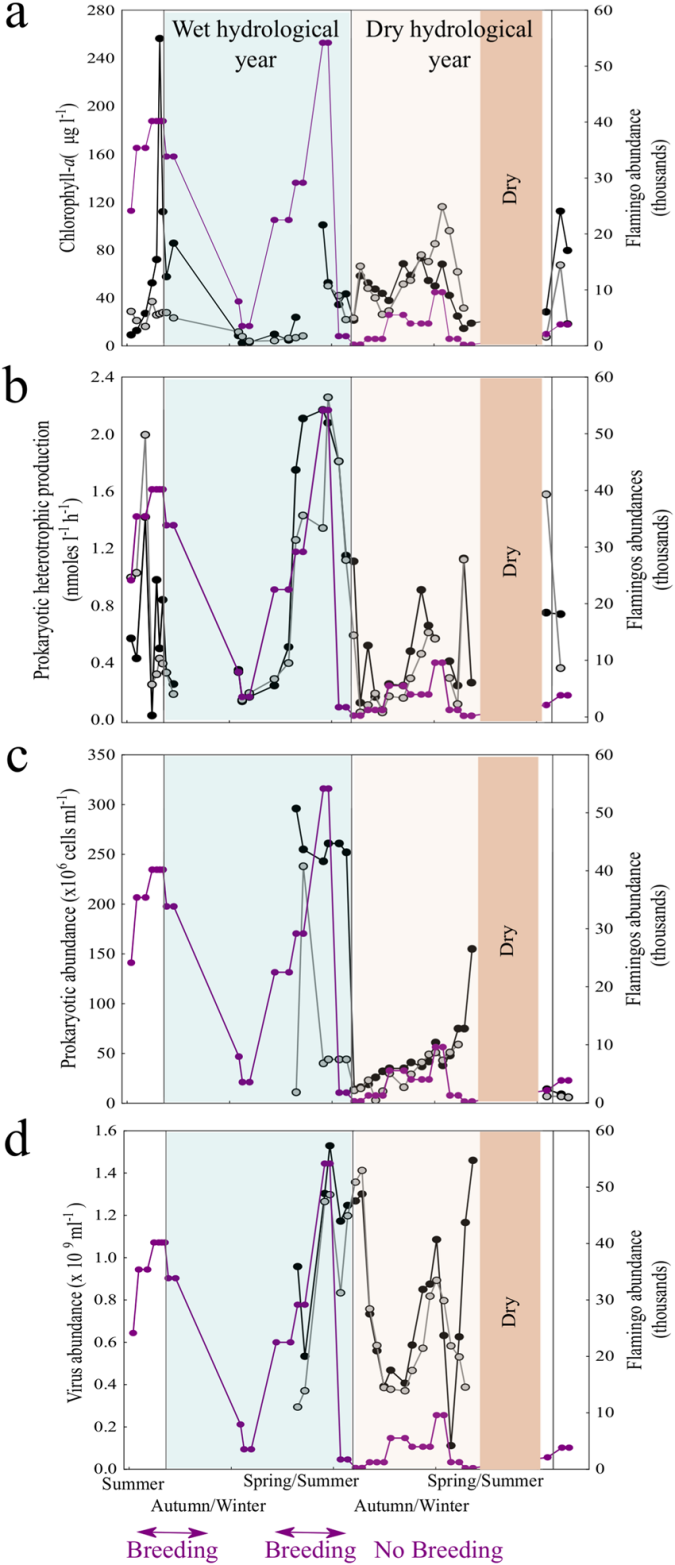
Microbial dynamics during two hydrological years. Changes in (**a**) chlorophyll *a* concentration, (**b**) prokaryotic heterotrophic production, (**c**) prokaryotic abundance, and (**d**) virus abundance at station 1 (black dots) and station 2 (grey dots). Flamingo abundance (purple line) is shown for reference.

Chlorophyll-*a* ranged over two orders of magnitude from 2 μg l^−1^ to 256 μg l^−1^. During the wet year at Station 1 (black dots), chlorophyll-*a* showed maximum values concurrent with two peaks in flamingo abundance (Fig. 4a). Indeed, in this station we observed a significant and positive correlation between the abundance of flamingos and chlorophyll-*a* concentration (n = 38, r = 0.34, p < 0.05). In contrast, during the dry year, when flamingos were less abundant, the highest values of chlorophyll-*a* were observed during the filling phase (lower salinity) in winter (Fig. 4a). Prokaryotic heterotrophic production (PHP) ranged 40 fold from 0.05 nmoles of leucine l^−1^h^−1^ to 2.25 nmoles of leucine l^−1^h^−1^ with maximum values synchronous and correlated with the peaks in flamingo abundance (Fig. 4b) at both Station 1 (black dots, n = 37, r = 0.48, p < 0.05) and Station 2 (grey dots, n = 36, r = 0.36, p < 0.05). Prokaryote abundance ranged over three orders of magnitude from 3 to 296 (×10^6^) cells ml^−1^ (Fig. 4c), while virus abundance ranged from 0.3 to 1.5 (×10^9^) particles ml^−1^ (Fig. 4d). The peak in the prokaryote abundance also coincided with the highest abundance of flamingos, while viral abundance (VA) was similar in both years. VA dynamics appeared time lagged with respect to the abundance of flamingos in the wet year. In contrast, in the dry year the maximum VA coincided with the highest salinity, during the evaporation phase (Fig. 4d). To explore in more detail the influence of flamingos and salinity on the abundance of viruses in the water column, we performed a multiple regression analysis (Table 3). Both variables significantly affected viral abundance, although the contribution of flamingos was slightly greater.

**Table 3:** Results of multiple regression analyses to assess the influence of flamingo abundance and salinity on the concentration of viruses in Fuente de Piedra lake.

Independent Variables	Virus abundance (ml^−1^)		
	*r*	*b*	p-*level*
Flamingos	0.548973	12.7	0.002028
Salinity (ppt)	0.506379	4634.3	0.004089
Intercept		243837.9	0.180274

The *r* values are partial correlation coefficients, *b* is the non-standardized regression coefficients.

### Cascading effects of flamingos

**Figure 5:**
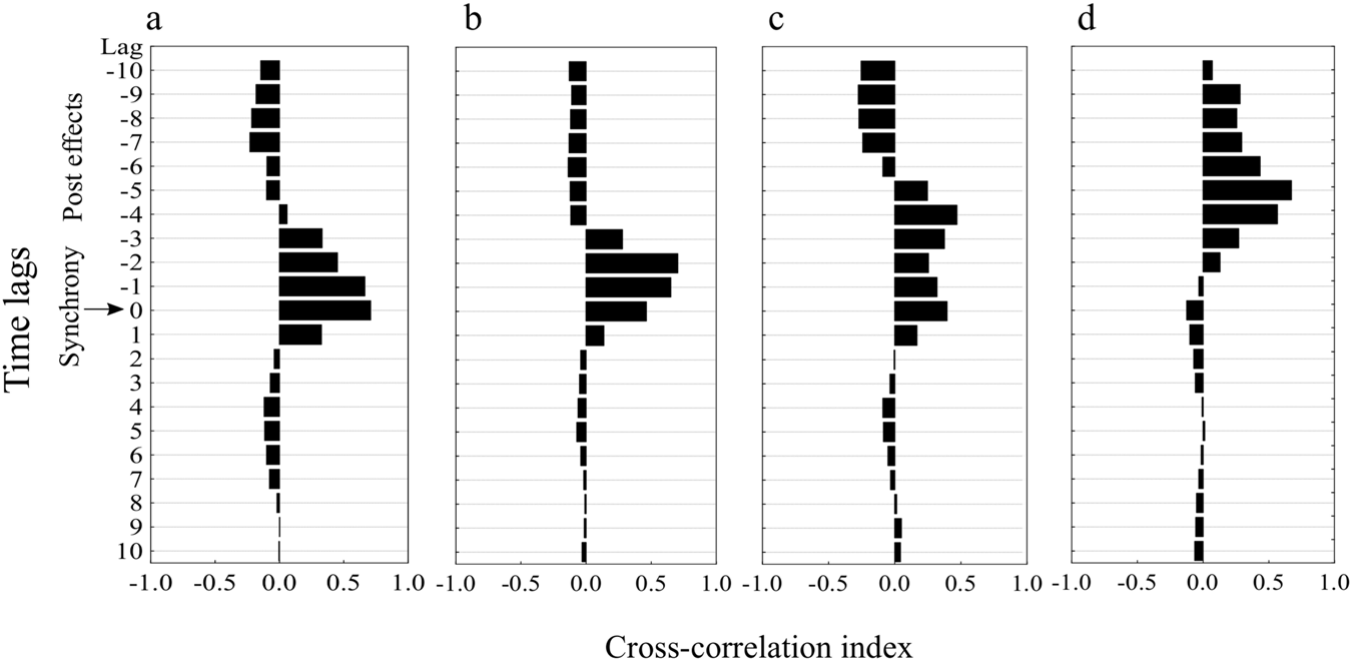
Cross-correlations of flamingos and the microbial and total dissolved nitrogen dynamics. The zero line represents a synchronous effect and the negative numbers represent time-lag effects at different intervals. The analyses were performed for (**a**) prokaryotic heterotrophic production, (**b**) prokaryotic heterotrophic abundance, (**c**) virus abundance and (**d**) total dissolved nitrogen. Significant positive effects are represented by black bars that exceed the 0.5 level on the x axis.

Prokaryotic heterotrophic production and abundance appeared to be synchronized with the abundance of flamingos (Fig. 4b and c), whereas the abundances of viruses (Fig. 4d) and total dissolved nitrogen concentration (Fig. 3f) showed different time lags. To explore the existence of potential links among these synchronous or time-lagged variables associated with flamingo abundance, we performed cross-correlation analyses. Flamingo abundance (y) was significantly cross-correlated with prokaryote activity (x) at lag = 0 (r_xy_ = 0.70) and lag = −1 (r_xy_ = 0.66) (Fig. 5a), with prokaryote abundance (x) at lag = 0 (r_xy_ = 0.46), lag = −1 (r_xy_ = 0.65) and lag = −2 (r_xy_ = 0.70) (Fig. 5b), and with total dissolved nitrogen (x) at lag = −4 (r_xy_ = 0.56) and lag = −5 (r_xy_ = 0.67) (Fig. 5d). Cross-correlations with virus abundance (x) were not significant, but the coefficient was highest at lag = −4 (Fig. 5c).

### Nutrients delivered by flamingo guano

The nutrient content of flamingo chick feces ranged from 28.7 to 45.1 mg N g^−1^ dry weight, and from 2.1 to 3.4 mg P g^−1^ dry weight depending on food sources (Table 4). Almost all the nitrogen in flamingo chick feces was soluble, with TDN averaging 74% of TN. In contrast, TDP only averaged 19% of TP (Table 3). The molar TN:TP ratio in flamingo chick feces was 29, above the Redfield ratio and higher than found in the feces of other waterbirds (Table 3). Assuming the average values of N and P content in flamingo droppings (i.e. TN = 36.9 mg N g^−1^ and TP = 2.75 mg P g^−1^ dry weight), we estimated that the average per capita delivery in the lake per day was 1.978 g N (141.2 mmol of N) and 0.147 g P (4746.52 μmol of P). Given the abundance of flamingos, the total delivery of N to the lake was of 16.54 tonnes during the wet year (i.e. 16.7 Kg N ha^−1^y^−1^) and 1.67 tonnes during the dry year (i.e. 3.2 Kg N ha^−1^ y^−1^). Taking into account the water volume, the annual N input during the wet year was 3.372 mg N l^−1^y^−1^, and during the dry year was 1.342 mg N l^−1^y^−1^. The total delivery of P to the lake was of 1.23 tonnes (i.e. 1.24 Kg P ha^−1^ y^−1^) during the wet year and 0.12 tonnes (i.e. 0.23 Kg P ha^−1^ y^−1^) during the dry year. The annual P input was 0.251 mg P l^−1^y^−1^ during the wet year, and 0.100 mg P l^−1^y^−1^ during the dry year.

**Table 4:** Means and standard deviations (±SD) of total nitrogen (TN), total dissolved nitrogen (TDN), total phosphorus (TP), total dissolved phosphorus (TDP), total organic carbon (TOC), and dissolved organic carbon (DOC) in mg per g of feces in dry weight and the molar TN: TP ratios in the feces of the greater flamingo (*Phoenicopterus roseus*) and other waterfowl.

Waterbirds	TN mg-N g^−1^ (mmol-N g^−1^)	TDN mg-N g^−1^ (mmol-N g^−1^)	TP mg-P g^−1^ (mmol-P g^−1^)	TDP mg-P g^−1^ (mmol-P g^−1^)	TOC mg-C g^−1^ (mmol-C g^−1^)	DOC mg-C g^−1^ (mmol-C g^−1^)	TN: TP (Molar)	Reference
*Phoenicopterus roseus* (brown feces) Soluble	28.78 ± 1.42 (2.05 ± 0.10)	24.83 ± 4.53 (1.77 ± 0.32) (86%)	2.06 ± 0.98 (0.07 ± 0.03)	0.45 ± 0.05 (0.01 ± 0.00) (19%)	125.77 ± 0.55 (10.48 ± 0.04)	106.97 ± 0.76 (8.91 ± 0.06)	29	This study
*Phoenicopterus roseus* (pink feces) Soluble	45.15 ± 1.80 (3.22 ± 0.12)	29.55 ± 5.41 (2.11 ± 0.38) (66%)	3.38 ± 0.37 (0.11 ± 0.01)	0.77 ± 0.03 (0.02 ± 0.00) (20%)	127.25 ± 3.15 (10.60 ± 0.26)	112.97 ± 0.14 (9.41 ± 0.01)	29	This study
*Branta leucopsis* Barnacle goose	11 ± 2 (0.8 ± 0.1)	—	3.3 ± 0.2 (0.1 ± 0.0)	—	450.5 ± 3.5 (37.5 ± 0.3)	—	7.5	Van Gueest *et al*.^13^
*Anous minutus Bo*. Black noddy	182.5 (13.03)	—	37.5 (1.21)	—	—	—	10.7	Smith & Johnson^80^
*Phalacrocorax carbo L*. Great cormorant	32.8 (2.3)	—	143.2 (4.6)	—	—	—	0.6	Marion *et al*.^81^
*Ardea cinerea L*. Grey heron	42.1 (3.0)	—	114.7 (3.7)	—	—	—	0.8	Marion *et al*.^81^
*Pelecanus thagus* Peruvian pelican	241.3 (17.2)	—	20.9 (0.7)	—	—	—	25	Hutchinson^1^

Two types of feces (brown and pink) were analyzed and are likely to be associated with different food sources. Dissolved nutrient solubilized from feces (TDN, and TDP) were calculated as the percentage (%) of the total (TN and TP).

### Experiments with flamingo guano

**Figure 6:**
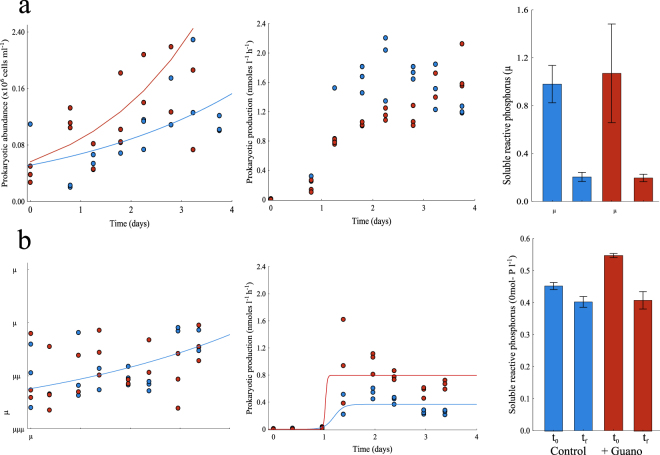
Experiments on the growth of heterotrophic prokaryotes with addition of flamingo guano. Changes in heterotrophic abundance and production during the period of incubation, and the concentration of soluble reactive phosphorus at the beginning (t_0_) and end (t_f_) of (**a**) experiment 1 and (**b**) experiment 2. Blue represents the treatment with the control conditions and red the treatment with addition of fresh guano from flamingo chicks.

We tested if nutrients delivered by guano directly promote microbial growth in two laboratory experiments (Fig. 6). In experiment 1 (Fig. 6a), prokaryote abundance in re-growth cultures fitted exponential growth. The specific growth rate (μ) in the treatment with guano was almost twice as high (μ = 0.46 ± 0.16 d^−1^; r^2^ = 0.39) as in the control treatment (μ = 0.27 ± 0.01 d^−1^, r^2^ = 0.35), a significant difference. Prokaryote heterotrophic production did not fit exponential or logistic growth curves. In experiment 2 (Fig. 6b), only the control treatment fitted exponential growth (μ = 0.21 ± 0.07 d^−1^, r^2^ = 0.30) and the specific growth rate (μ) was similar to the control in the previous experiment. Prokaryote heterotrophic production, however, fitted logistic growth curves in both treatments. Both the carrying capacity (K) and the specific growth rates for protein synthesis (b) were higher in the treatment with guano (K = 0.797 nmoles l^−1^h^−1^, b = 295 d^−1^, r^2 = ^0.72) than in the control treatment (K = 0.366 nmoles l^−1^h^−1^, b = 11 d^−1^, r^2 = ^0.71). At the beginning and end of the experiments, we also monitored the changes in dissolved nutrients. DOC and TDN did not change significantly over the incubation period in any of the experimental treatments (Table S1). However, SRP decreased significantly in all treatments at the end of the incubation time (Fig. 6a and b), likely due to its assimilation by heterotrophic prokaryotes during their growth, indicating a high P-demand by these microorganisms.

## Discussion

We observed a robust, direct and synchronous influence of flamingos on heterotrophic prokaryotic production and abundance, that appears to be mediated by an increase in P availability associated with inputs from guano and sediment bioturbation, instead of by organic substrates derived from primary producers. This direct influence of flamingos on heterotrophic prokaryotic activity appears to trigger cascading effects on heterotrophic prokaryotes, virus abundance and total dissolved nitrogen. The addition of feces and sediment bioturbation when the water level was low affected the availability of dissolved phosphorus and nitrogen, whereas DOC, total nitrogen and total phosphorus were mainly driven by the hydrological phases (evaporation vs. precipitation).

In Fuente de Piedra Lake, heterotrophic prokaryotic production and primary production (with chlorophyll-*a* as a proxy) were both affected by the abundance of flamingos. Similar results on primary production were reported by Kitchell *et al*.^3^, who showed a positive correlation between geese density and chlorophyll-*a* concentrations, which increased eight-fold in ponds with high bird densities. Chlorophyll-*a* increases have been linked to N and P inputs from waterbird feces^2,6^, and also experimentally demonstrated^13^. In our study, this effect was even more dramatic at Station 1, where chlorophyll-*a* increased by up to 25 times during the flamingo-breeding season. On the other hand, prokaryotic heterotrophic production was synchronously correlated to flamingo abundance at both sampling stations (Fig. 4b) and to total dissolved phosphorus (Fig. S2). Different mechanisms could explain these synchronous dynamics between heterotrophic prokaryotes and flamingo abundance (Fig. 4b and c). On the one hand, heterotrophic prokaryotes could have been indirectly stimulated via phytoplankton exudation of organic carbon, which responded to the nutrients delivered by flamingos. On the other hand, flamingos may have a direct effect on prokaryotes through the inputs of limiting nutrients via guano deposition and sediment bioturbation as they move. The first possibility seems unlikely in this study since the relationship between flamingo abundance and chlorophyll-a was only significant in one of the stations and was weaker than the relationship between flamingo abundance and the PHP and prokaryote abundance. In fact, we found a positive and significant correlation between prokaryotic heterotrophic production and TDP during the wet year when flamingos reached maximum abundance (n = 39, r = 0.39, p < 0.01, Fig. S2 in supplementary material), suggesting P-limitation of the heterotrophic prokaryotes during this period.

The experiments with guano addition clearly confirmed a stimulus of prokaryote growth coupled with a significant consumption of soluble phosphorus and not other nutrients during the incubation (Fig. 6, Table S2). This confirms that the interaction between flamingos and prokaryotes was mediated by soluble phosphorus. Usually bacteria outcompete primary producers for inorganic nutrients, especially in P-limited systems^53–55^. Mindl *et al*.^56^ observed a similar response in wetlands in the Arctic, where bacterial production and diversity increased when geese were present.

The nutrient inputs associated with the flamingo colony can be either from fecal inputs during breeding^4–6,57^ or from sediment bioturbation due to wading and foraging activity^58,59^. Sediment bioturbation by waterbirds, particularly flamingos, plays a crucial role in the functioning of these wetlands^14^. In fact, there was a linear increase of TDP as water level dropped below 80 cm (Fig. S1). This is the maximum depth at which flamingos wade^60^. Previous studies^61^ have reported high SRP concentration in the interstitial water of sediments from Fuente de Piedra lake. In fact, SRP concentration was more affected by water level than by the flamingo abundance itself (Table 2), suggesting a major influence of sediments on SRP concentration in the water column of Fuente de Piedra. SRP dynamics in this system is complex and also affected by wave action and by physicochemical processes in sediments, such as adsorption/absorption, precipitation, solubilisation and redox reactions. Clavero *et al*.^62^ performed several experiments in this lake showing that phosphate release from sediments was stimulated at salinities above 70 ppt. Therefore, the dissolved phosphorus in the system results from a combination of flamingo aggregations with fecal inputs and sediment bioturbation, along with an increase in the influence of sediments during the evaporation phase.

On the other hand, the fecal inputs from flamingo chicks contained comparatively more nitrogen than phosphorus with N:P molar ratios above the Redfield ratio (Table 3). These high ratios have several potential explanations which are not mutually exclusive: the feeding of chicks with N-rich food, efficient P retention by the chicks, the a wide-range of N:P ratios among waterbird feces, and the use of fresh, not dried, feces in this study. Flamingo adults make regular movements during breeding from the Fuente de Piedra colony to nitrogen-enriched feeding sites such as rice fields and fishponds in the Doñana wetland complex^31,32^. Our data for flamingo feces were similar to those of Hutchinson^1^ for the Peruvian pelican that has a fish diet rich in proteins. We collected the chick feces during ringing operations and they were immediately frozen, preventing significant N losses related to ammonium volatilization, a common process in nature where feces are deposited and exposed to the atmosphere for days^63,64^. Finally, we were only able to sample feces from growing chicks, and it is possible that adult flamingos have feces with a lower N:P ratio, since cloacal microbiota appears to differ with age^65^ and chick retain P^66^ which could modify the N:P ratio in feces. However, the evidence from this study suggests flamingo fecal inputs influence also the dissolved nitrogen in the study lake (Figs 3f, 5d).

Previous studies have emphasized the importance of waterbirds as nutrient vectors in aquatic ecosystems with high water retention times in arid and semiarid regions^8,9^. However, in Fuente de Piedra Lake, total N and P inputs were more than 5-fold higher during the wet year than in the dry year. During the wet year, the flamingo population size was substantially larger than in the dry year, as were their corresponding N and P inputs (16.7 Kg N ha^−1^ y^−1^ and 1.24 Kg P ha^−1^y^−1^). These values were higher than those reported for herbivorous (1.07 kg N ha^−1^ y^−1^ and 0.10 kg P ha^−1^ y^−1^) or carnivorous (0.26-0.65 kg N ha^−1^ y^−1^ and 0.12-0.16 Kg P ha^−1^ y^−1^) waterbirds in the Netherlands^4,5^, or in Lake Mattamuskeet (0.2 kg N ha^−1^ y^−1^) in North Carolina^67^. On the other hand, these allochthonous inputs of N and P from flamingo guano were higher than the atmospheric inputs of 5.89 Kg N ha^−1^ y^−1^ and 0.18 Kg P ha^−1^y^−1^ reported for the study region^68^. These results underline the importance of waterbirds as nutrient vectors and how extreme conditions, such as the drying of the lake and the resulting crash in the numbers of flamingos, can reduce them.

The links between dissolved nutrients and flamingo abundance, however, do not appear to be simple. TDN was time lagged with flamingo abundance (Figs 3f, 5d) and was also affected by water level (Table 2). This time lagged correlation between TDN and flamingo abundance could be partially related to a cascading effect of flamingos on the microbial community (Fig. 5). The high flamingo abundance during the breeding season stimulates heterotrophic prokaryotic activity, which leads to an increase in the heterotrophic prokaryotic abundance with a subtle time lag (Fig. 5a,b). Higher bacterial abundance facilitates contact between viruses and prokaryotes and may enhance bacterial lysis, leading to more viruses (Fig. 5c) and the release of nutrients (Fig. 5d). There is evidence that viruses affect heterotrophic prokaryote mortality in saline systems^69^, and that viral lysis releases soluble nitrogen and phosphorus^70,71^. Then, TDN concentration, with a time lag relative to the peak of virus abundance (Fig. 5d), may partially be a by-product of viral lysis. Unlike the dissolved P that is quickly incorporated by prokaryotes, the non-limiting nutrients, such as dissolved nitrogen, might be retained longer in the water column and be detected at higher concentrations. However, despite the absence of N-limitation in the studied system, TDN concentration decreased during the evaporation phase of the dry year (Fig. 3f), presumably as the result of other microbial processes such as denitrification, N_2_O production or ammonium volatilization. These processes seem to be promoted in sites with important inputs of N from wastewater, agricultural lands or atmospheric deposition^72–74^. Since wetlands are considered efficient ecosystems for removing nitrogen and improving water quality^75^, the influence of waterbirds on these processes of N loss should also be included in future studies. In fact, Winton & Richardson^76^ recently found that overgrazing by waterfowl had consequences for methane and nitrous oxide emissions. Waterfowl herbivory led to increases in methane emissions but prevented nitrification.

In this study, we demonstrated that flamingos stimulated the production of heterotrophic prokaryotes and produced a series of cascading effects on virus abundance and dissolved nitrogen. This stimulus was related to the inputs of dissolved nutrients from feces and sediments. Soluble phosphorus from feces was quickly incorporated into prokaryote biomass in experiments, and sediments appear to release soluble P at high salinity when water levels are low, allowing flamingo wading. The comparatively high inputs of N delivered by flamingos appeared to be time-lagged, but the processes involved in this lag, and particularly in its subsequent loss, are complex; other microbial processes such as ammonium volatilization, denitrification and nitrous oxide emissions should be considered in future research. N and P inputs by flamingos were higher during the wet hydrological year than during the dry hydrological year, suggesting that extreme conditions such as drought control the abundance of flamingos in this system and consequently their effects on dissolved nutrients and microbial production.

Overall, the study lake appears to have two levels of control of nutrients and microbial dynamics. At the ecosystem level, the climatic conditions (rainfall and environmental temperature) determine the hydrological budget, affecting the evapo-concentration of total nutrients (TN and TP), dissolved organic carbon, the water level and the abundance and breeding of flamingos. At a second level, there is a flamingo top-down control on dissolved nutrients (SRP and TDP) by inputs of guano and sediment bioturbation. These dissolved nutrients affect primary and heterotrophic production. Flamingos provide a major ecosystem service to humans through their cultural value^77^ and here we show that they also provide an ecosystem service by providing bioavailable nutrients that boost microbial production directly. Microbial activity facilitates the processing of organic carbon, and probably organic nitrogen, improving water quality in wetlands^75^. Nevertheless, further work should explore shifts in the composition of the microbial communities and functional groups to unravel the impact of flamingos and weather patterns on wetlands functioning and to obtain more precise information with predictive value.

Future scenarios of intense droughts in the Mediterranean biome could lead to increases in total nutrients in wetlands by evapo-concentration, but the biological (autotrophic and heterotrophic) activity might be constrained by the availability of dissolved phosphorus and nitrogen, which is more dependent on the water level and presence of waterbirds. Here, we show that flamingo abundance decreased during the dry hydrological year, as did the primary and the microbial production. These facts suggest, unlike our initial hypothesis, a more active ecosystem during the wet periods than during the dry ones. Droughts can reduce biological activity in this type of Mediterranean wetland, although in less extreme systems waterbird overcrowding could occur leading to acute guanotrophication. The consequences of waterbird overgrazing on biogeochemical cycles and other key processes in wetlands, such as greenhouse gas emissions, are still controversial^76,78,79^, and more field and experimental studies are needed to determine the lake-specific conditions that minimize the emissions of greenhouse gases while maintaining waterbird populations. Therefore, an integrated approach to wetlands management should determine the carrying capacity for each system, so that it houses waterbirds that promote denitrification to N_2_, but avoids the negative effects on water quality by guanotrophication, and potential increases in methane and nitrous oxide emissions.

## Electronic supplementary material

Supplementary information
