# Supplementary material for: Flamingos and drought as drivers of nutrients and microbial dynamics in a saline lake
Source: Sci Rep. 2017 Sep 22;7:12173. doi: 10.1038/s41598-017-12462-9 (PMC5610251; doi:10.1038/s41598-017-12462-9)
Supplement: Supplementary file 1 — Supplementary information [file 41598_2017_12462_MOESM1_ESM.pdf]

Flamingos and drought as drivers of nutrients and microbial dynamics in a saline lake

Gema L. Batanero[1], Elizabeth León-Palmero[1], Linlin Li[2], Andy J. Green[3], Manuel Rendón-Martos[4], Curtis A. Suttle[5], Isabel Reche[1*]

[1] Departamento de Ecología e Instituto del Agua, Universidad de Granada, 18071 Granada, Spain

[2] Department of Natural Resources, Faculty of Geo-information Science and Earth Observation, University of Twente, 7500 AE Enschede, The Netherlands

[3] Departamento de Ecología de Humedales, Estación Biológica de Doñana, CSIC, 41092 Sevilla, Spain

[4] Reserva Natural Laguna de Fuente de Piedra, Consejería de Medio Ambiente y Ordenación del Territorio, Junta de Andalucía, Apartado 1, 29520 Fuente de Piedra, Málaga, Spain

[5] Departments of Earth, Ocean & Atmospheric Sciences, Microbiology & Immunology, and Botany, and the Institute for the Oceans and Fisheries, University of British Columbia, Vancouver, BC Canada V6T 1Z4.

*Corresponding author:

Isabel Reche

Departamento de Ecología, Universidad de Granada, 18071 Granada, Spain

E-mail: ireche@ugr.es

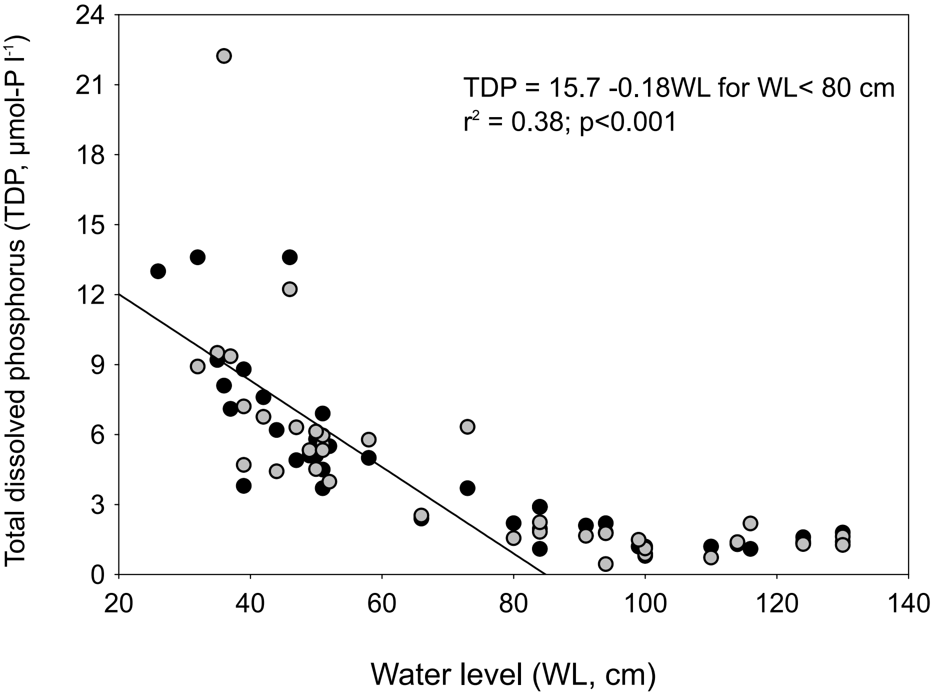

Figure S1 Linear negative relationship between the total dissolved phosphorus and the water level for depths lower than 80 cm

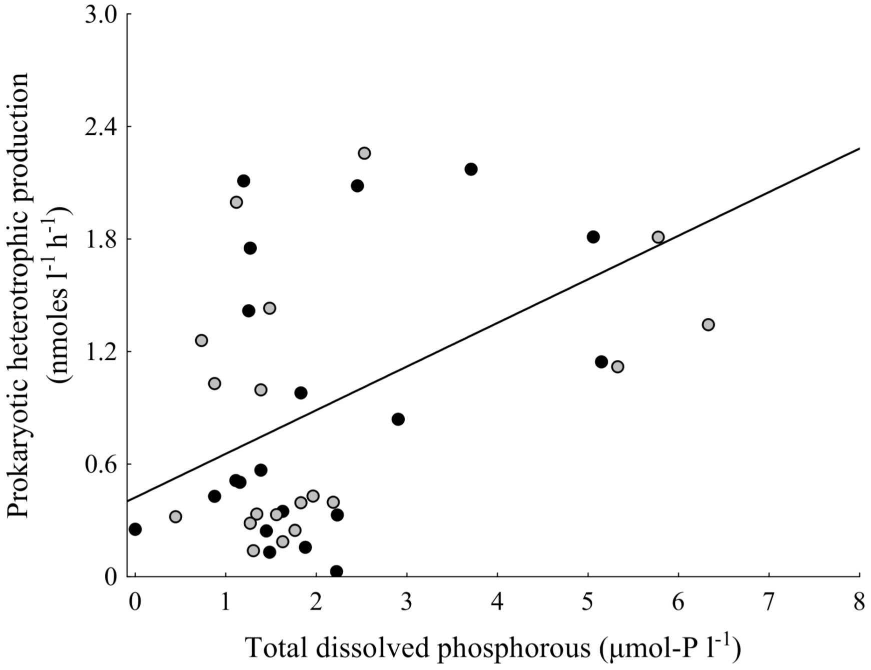

Figure S2 Relationship between total dissolved phosphorus and prokaryotic heterotrophic production during the wet cycle. Black dots represent data for station 1 and grey dots for station 2.

Table S1 Details of two guano addition experiments. The water level and flamingo abundance in the lake is shown for the time the experiments were performed. Results are presented for the concentrations of dissolved organic carbon (DOC) and total dissolved nitrogen (TDN) at the beginning (t_0) and end (t_f) of the experiments.

Water level (cm)	Flamingo abundance	Treatment	DOC (mmol-C l^{-1})		TDN (mmol-N l^{-1})	
79	33849	Exp. 1	t_0	t_f	t_0	t_f
		Control	2.00± 0.02	2.21 ± 0.12	0.20 ± 0.00	0.18 ± 0.00
		+ Guano	1.94 ± 0.05	2.09 ± 0.15	0.27 ± 0.00	0.24 ± 0.00
130	22490	Exp. 2	t_0	t_f	t_0	t_f
		Control	1.17 ± 0.09	1.12 ± 0.03	0.07 ± 0.00	0.07 ± 0.01
		+ Guano	1.26 ± 0.02	1.19 ± 0.10	0.09 ± 0.01	0.09 ± 0.01
